# The Perceived Impact of COVID-19 on Functional Activities Among Canadian Education Workers: A Cross-Sectional Study

**DOI:** 10.3389/fpubh.2022.879141

**Published:** 2022-06-27

**Authors:** Frances Serrano, Behdin Nowrouzi-Kia, Bruce Oddson, Rita Bishai, Jennifer Casole, Basem Gohar

**Affiliations:** ^1^Department of Psychology, University of Guelph, Guelph, ON, Canada; ^2^Department of Occupational Science and Occupational Therapy, Temerty Faculty of Medicine, The University of Toronto, Toronto, ON, Canada; ^3^Centre for Research in Occupational Safety and Health, Laurentian University, Sudbury, ON, Canada; ^4^School of Kinesiology and Health Sciences, Laurentian University, Sudbury, ON, Canada; ^5^Department of Psychology, University of Wilfred Laurier, Waterloo, ON, Canada; ^6^Department of Special Education, Loretto College, Toronto, ON, Canada; ^7^Department of Population Medicine, University of Guelph, Guelph, ON, Canada

**Keywords:** COVID-19, functional activities, perceived impact, education workers, cross-sectional

## Abstract

**Objective:**

This cross-sectional study examined the self-perceived impact of the COVID-19 pandemic on 2,378 education workers in Ontario, Canada, during the second wave.

**Methods:**

We examined six domains of functioning as per the short version of the World Health Organization Disability Assessment Schedule-2.0. Participants selected if their functioning had improved, remained unchanged or worsened during the pandemic for each item.

**Results:**

Educational workers described a general worsening of functional activities since the beginning of the pandemic. Moderate-to-extreme challenges were reported for all six functional domains. These challenges appeared to aggravate functional challenges for workers with disability, as indicated by pre-existing work accommodations. Older participants reported worse mobility than younger participants; however, they appeared to have better coping skills in learning new tasks and maintaining friendships. Women were more likely to report difficulties in maintaining household responsibilities.

**Conclusions:**

We consider the role of mental health challenges and pre-existing inequality as predictors of pandemic-related difficulties. Recommendations include more longitudinal research in this population and policymakers to incorporate a health promotion lens to support their education workers more proactively.

## Introduction

Education workers, including teachers, educational assistants, and other support staff, have highly demanding jobs characterized by long working hours, heavy workloads, and emotional demands (1). These working conditions take a toll, and as a profession, teachers are known to have comparatively poor physical health and psychological wellbeing (2, 3). This matters in several ways. The health challenges of education workers may be difficult to navigate in themselves, potentially leading to high levels of absenteeism (4) and leaving the profession. Employers may find it challenging to meet their responsibilities for workplace health when the general level of distress is high. Finally, educational workers are central in the care of children. The difficulties faced by education workers may, in turn pose greater challenges to meet their needs.

### The Impact of COVID-19

The COVID-19 pandemic had a significant impact on people worldwide (5). In most areas, significant public health measures were imposed to reduce the spread of the virus, such as closures of various businesses, including fitness centers and limits on the number of visitors in a household (6). These measures reduced the amount and quality of social interactions and added challenges in maintaining quality of life (7). The restrictions and their indirect consequences disrupted daily functions such as socialization, exercise, sleep, and healthy eating behaviors. In addition, recent studies have highlighted the pandemic's adverse impact on the general population's mental health, resulting in frustration, stress, and depression (5, 8). These undesirable outcomes may have been exacerbated in individuals with pre-existing disabilities due to reduced access to care, physical activities, and mood changes (9). These general results raise a concern about education workers since their background levels of stress and functional impairments may interact with the challenges generated by our response to COVID-19.

Canadian education workers may differ from those in other countries in several ways, for example, due to differences in their work environments and stability of employment. However, like those in other countries, Canadian education workers made rapid and significant changes in how they provided services. Moreover, given their pre-existing high prevalence of psychological distress and impaired functional activities the impact of COVID-19 is of particular concern.

To our knowledge, this is the first Canadian study that assessed the perceived impact of the pandemic and associated public health measures on the level of disability and functional challenges faced by education works in the province of Ontario, Canada.

## Materials and Methods

The study was approved by the University of Guelph's Research Ethics Board (REB# 20-06-002). This prospective cross-sectional study is a part of a larger undertaking that examined the impacts of the pandemic on Ontarian education workers. We used the STROBE checklist to ensure quality and accuracy when preparing this study (10). We examined the functional activities of education workers across Ontario, Canada, during the second pandemic wave, which began in in Ontario in the fall of 2020. The survey was disseminated between October 2020 and January 2021 *via* Qualtrics (11), with one follow-up email sent in December 2020. At the time of this study, Ontarian education workers were asked to return physically to the workplace following school closures in the spring of 2020 until the summer holidays. In some schools, teachers used a hybrid teaching model where they simultaneously taught students in person and others virtually.

We define education workers as unionized employees in the public education sector ranging from kindergarten to secondary. They include teachers, educational assistants, supply teachers, early childhood educators, administrative staff, and support workers who provide specialized services, including psychology, social work, and communicative supports. Eligible participants included those employed during the first wave of the pandemic and have returned to work during the second wave. We partnered with provincial unions, who agreed to disseminate the questionnaire on our behalf. Specifically, the survey links were disseminated from the executive to the district levels. Next, district leaders disseminated the survey links to their local members.

Participation was purely voluntary, and our anonymous survey could be completed in either English or French. Informed consent was obtained at the beginning of the survey. We collected demographic information, including age, gender, marital status, occupational groups, and employment status (i.e., permeant vs. contract, part-time vs. full-time). Participants also identified if they received accommodations from their employer due to physical or psychological disability.

### Questionnaire

The World Health Organization Disability Assessment Schedule 2.0 (WHODAS 2.0 SF) is a 12-item self-rated health questionnaire that assesses the behavioral limitations and restrictions to participation experienced by individuals independent of a medical diagnosis in the past 30 days (12, 13). Items are scored on a 5-point Likert-type scale ranging from “none” to “extreme or cannot do.” The WHODAS 2.0 SF has shown robust psychometric properties (9, 13). It has a test-retest reliability of 0.93–0.96 at the domain level and good internal consistency (Cronbach's alpha ≥ 0.81). Papadopoulou et al. (12) found strong intraclass correlation (ICC = 0.99; *p* < 0.001), suggesting excellent reliability. Their results also suggest strong construct and convergent validity (12).

The WHODAS 2.0 was recently used to assess the psychosocial wellbeing in the workplace during the pandemic (14). We used the WHODAS 2.0 to guide our survey of changes to functions during the period of accommodating the work changes and stresses imposed by COVID-19 and associated health measures. Specifically, we asked about participants' (1) cognition, (2) mobility, (3) self-care, (4) getting along, (5) life activities, and (6) participation. Each domain consists of two items. The cognition domain asks about learning new tasks and concentration. Mobility explores one's ability to stand for longer than 30 min and walking long distances. Self-care includes items on body washing and the ability to get dressed. Getting along focuses on how people deal with others and their ability in maintaining friendships. Life activities explores the ability to complete household responsibilities and day-to-day work. Finally, participation explores the ability to join group activities and how one is emotionally affected by health problems. In addition, for each question, a follow-up asked participants to rate whether, since COVID-19, their response has improved, stayed the same or worsened.

### Statistical Analyses

Descriptive statistics and cross-tabulations for the WHODAS 2.0 SF items were used to describe the background level of functioning in this sample and investigate the overall level of perceived impact of COVID-19.

To investigate the relationships between pre-existing functional difficulties, demographic predictors, and the perceived impact of COVID-19, we conducted stepwise binary logistic regressions at the item level.

Goodness of fit was assessed using Hosmer-Lemeshow for each analysis. Additionally, multicollinearity was assessed using the tolerance threshold and Variance Inflation Factor (VIF). The models are expressed in odds ratios (OR) and corresponding 95% confidence intervals (CI).

We dichotomized the WHODAS items into two categories (1 = “none to mild”; 2 = “moderate to extreme”). Deciding on this split was determined by the research team's clinicians (occupational therapist and psychologist) in consultations with the team's statistician. Superficially, we believe that participants experiencing moderate severity levels or higher on any of the WHODAS items is of clinical concern. Furthermore, we dichotomized age as a predictor variable since the sample was evenly split between those below and above the mean age (<45 and ≥45). As a *post-hoc* analysis, we also examined age as a continuous variable to determine if there is a linear relationship. For the regression models, we included only binary gender responses (“man” or “woman”). Approximately 0.5% (*n* = 13) identified as “non-binary” or “other,” and only 1% (*n* = 25) chose not to respond. The need for accommodations was also conceptualized in two levels (“no” or “yes”). Finally, the perceived impact of COVID-19 on each WHODAS item had three levels (‘better than,” “the same as,” or “worse than” before the pandemic). The first level of each variable served as the referent group except for the perceived impact of COVID-19 on the WHODAS items where “the same as” served as the referent group. All statistical tests were performed using SPSS 28.0 for Mac (15). Statistical significance was determined at the 0.05 level.

## Results

### Study Respondents

A total of 4,394 education workers completed the survey. Of those, 2,378 (54.1%) had sufficient information for data interpretation. The sample ranged from 18 to 81 years old (M = 44.82; SD = 9.163). Most participants identified as women (81.1%; *n* = 1,928), married, common law or in a committed relationship (75.4%; *n* = 1,794). Almost 87% of the sample comprised teachers, and over 85% were permanent, full-time employees. Approximately 8.4% (*n* = 199) required accommodations at work. Please see Table 1. The sample's characteristics are consistent with the population's characteristics.

**Table 1:** Demographic and job characteristics of the sample.

**Characteristic**	***n***	**%**
**Age** (*min*. = 18.0, *max*. = 81.0; *M* = 44.82; *SD* = 9.163)		
Below 45	1,131	47.6
45 or older	1,195	50.3
Missing	52	2.2
**Identified gender**		
Man	413	17.4
Woman	1,928	81.1
Non-binary or other	9	0.5
Choose not to answer	25	1.1
Missing	3	0.1
**Marital status**		
Married/common law/committed relationship	1,794	75.4
Separated/divorced	172	7.2
Single	333	14.0
Widowed	24	1.0
Choose not to answer	49	2.1
Missing	6	0.3
**Requiring accommodations**		
No	2,100	88.3
Yes	199	8.4
Missing	79	3.3
**Job classification**		
Teacher (including special education)	1,995	83.9
Occasional teacher/substitute teacher	63	2.6
Computer/technician/IT	4	0.2
Clerical/office	43	1.8
Education assistant	105	4.4
Maintenance/custodial	2	0.1
Early childhood educator/child and youth counselors	87	3.7
Psychological staff/social worker/speech and language pathologist/occupational therapist	31	1.3
Other	44	1.9
Missing	4	0.2
**Work schedule**		
Permanent full-time	2,131	89.6
Permanent part-time	94	4.0
Temporary full-time	105	4.4
Temporary part-time	47	2.0
Missing	1	0.04

*n, number of respondents per characteristic*.

Results from the cross-tabulation suggest a perceived decline in functional activities since the pandemic (Table 2). For instance, over 54% of the sample indicated moderate-to-extreme difficulties in their abilities to complete day-to-day work, with almost 69% reporting that this has worsened since the pandemic. Similar concerns were seen with joining community activities, being affected by other health problems, and concentrating on tasks for 10 min.

**Table 2:** Cross-tabulation of dichotomized WHODAS 2.0 scores and COVID-19 indicator.

**WHODAS items**	**WHODAS score**	**Since COVID-19, my response is _____ before**		
***In the past 30 days, how much difficulty did you have in…***				
		**Better than** ***n* (%)**	**The same as** ***n* (%)**	**Worse than** ***n* (%)**
1. Standing for long periods such as 30 min	*N* = 2,228	60 (2.7)	1,618 (72.6)	550 (24.7)
	None-to-mild	54	1,491	267
	Moderate-to-extreme	6	127	283
2. Taking care of household responsibilities?	*N* = 2,229	61 (2.7)	744 (33.4)	1,426 (63.9)
	None-to-mild	43	593	352
	Moderate-to-extreme	18	151	1,074
3. Learning a new task (e.g., how to get to a new place)?	*N* = 2,216	34 (1.5)	1,271 (57.4)	911 (41.1)
	None-to-mild	27	1,179	387
	Moderate-to-extreme	7	92	524
4. Joining in community activities?	*N* = 2,224	21 (.9)	755 (34)	1,449 (65.1)
	None-to-mild	13	650	490
	Moderate-to-extreme	8	105	959
5. Emotionally affected by other health problems?	*N* = 2,222	29 (1.3)	753 (33.9)	1,440 (64.8)
	None-to-mild	20	656	1,102
	Moderate-to-extreme	9	97	1,120
6. Concentrating on doing something for 10 min?	*N* = 2,216	42 (1.9)	1,085 (49)	1,089 (49.1)
	None-to-mild	36	1,007	465
	Moderate-to-extreme	6	78	624
7. Walking long distance such as a kilometer (or equivalent)?	*N* = 2,214	132 (6)	1,570 (70.9)	512 (23.1)
	None-to-mild	13	1,473	1,848
	Moderate-to-extreme	132	1,570	366
8. Washing your whole body?	*N* = 2,209	49 (2)	1,848 (84)	312 (14)
	None-to-mild	47	1,824	199
	Moderate-to-extreme	2	24	113
9. Difficulty getting dressed?	*N* = 2,202	51 (2.3)	1,789 (81.3)	362 (16.4)
	None-to-mild	51	1,764	265
	Moderate-to-extreme	0	25	97
10. Dealing with people you don't know?	*N* = 2,212	50 (2.3)	1,128 (51)	1,034 (46.7)
	None-to-mild	45	1,057	478
	Moderate-to-extreme	5	71	556
11. Maintaining friendship?	*N* = 2,212	47 (2.1)	1,009 (45.6)	1,156 (52.3)
	None-to-mild	42	942	564
	Moderate-to-extreme	5	67	592
12. Your day-to-day work?	*N* = 2,214	41 (1.9)	532 (24)	1,641 (74.1)
	None to mild	30	466	515
	Moderate-to-extreme	11	66	1,126

*N, Total number of respondents per item; n, number of respondents based on COVID-19 Indicator per item*.

### Predictors of Functional Activities

The Hosmer-Lemeshow test revealed a good fit with the logistic regression models (*p* > 0.05). Also, the assumption of linearity was not violated, and there was no presence of multicollinearity between variables (Tolerance > 0.1; VIF < 10). Table 3 depicts the adjusted ORs for each item.

**Table 3:** Logistic regressions for reporting worsened WHODAS 2.0 domains with explanatory variables of age, gender, and requiring accommodations during COVID-19.

**Variable**	**OR**	**95% CI** **(lower-upper)**	***P*-value**
**Domain 1: cognition**			
Learning new tasks			
Age	0.76	0.60–0.96	0.019^*^
Gender	1.24	0.91–1.71	0.177
Accommodations	1.29	0.87–1.91	0.211
Perception: better since COVID	2.71	1.09–6.76	0.032^*^
Perception: worse since COVID	17.46	13.46–22.62	<0.001^***^
Concentration			
Age	0.90	0.72–1.12	0.325
Gender	1.01	0.76–1.135	0.942
Accommodations	2.10	1.44–3.07	<0.001^***^
Perception: better since COVID	2.27	0.92–5.61	0.08
Perception: worse since COVID	18.45	14.03–24.27	<0.001^***^
**Domain 2: mobility**			
Standing for long periods			
Age	1.55	1.19–2.00	0.001^***^
Gender	1.40	0.98–1.99	0.062
Accommodations	2.32	1.56–3.44	<0.001^***^
Perception: better since COVID	1.33	0.56–3.19	0.522
Perception: worse since COVID	12.69	9.78–16.48	<0.001^***^
Walking long distances			
Age	1.59	1.20–2.09	0.001^***^
Gender	1.16	0.80–1.67	0.429
Accommodations	3.33	2.24–4.95	<0.001^***^
Perception: better since COVID	1.69	0.91–3.14	0.099
Perception: worse since COVID	14.48	10.90–19.23	<0.001^***^
**Domain 3: self-care**			
Washing the whole body			
Age	0.96	0.63–1.47	0.848
Gender	1.44	0.80–2.59	0.226
Accommodations	1.97	1.10–3.55	0.024^*^
Perception: better since COVID	3.94	0.89–17.42	0.071
Perception: worse since COVID	47.82	28.83–79.32	<0.001^***^
Getting dressed			
Age	1.41	0.92–2.17	0.118
Gender	1.13	0.62–2.06	0.683
Accommodations	1.75	0.97–3.16	0.063
Perception: better since COVID	N/A	0.00	0.998
Perception: worse since COVID	29.25	17.78–48.13	<0.001^***^
**Domain 4: getting along**			
Dealing with people don't know			
Age	0.96	0.76–1.20	0.714
Gender	1.00	0.75–1.34	0.995
Accommodations	1.88	1.28 −2.75	0.001^***^
Perception: better since COVID	1.77	0.68–4.62	0.245
Perception: worse since COVID	17.46	13.19–23.12	<0.001^***^
Maintaining friendships			
Age	0.67	0.54–0.84	<0.001^***^
Gender	1.18	0.89–1.56	0.257
Accommodations	1.34	0.92–1.94	0.125
Perception: better since COVID	1.88	0.71–4.96	0.202
Perception: worse since COVID	14.35	10.81–19.04	<0.001^***^
**Domain 5: life activities**			
Household responsibilities			
Age	0.76	0.62–0.94	0.10
Gender	1.68	1.29–2.19	<0.001^***^
Accommodations	1.67	1.14–2.43	0.008^**^
Perception: better since COVID	1.59	0.88–2.90	0.127
Perception: worse since COVID	11.67	9.33–14.6	<0.001^***^
Day-to-day work			
Age	0.75	0.61–0.91	0.004^**^
Gender	0.82	0.63–1.07	0.137
Accommodations	1.57	1.09–2.26	0.017^*^
Perception: better since COVID	2.49	1.15–5.39	0.020^*^
Perception: worse since COVID	15.61	11.68–20.85	<0.001^***^
**Domain 6: participation**			
Joining community activities			
Age	0.86	0.70–1.04	0.125
Gender	1.20	0.93–1.55	0.169
Accommodations	1.66	1.16–2.38	0.006^**^
Perception: better since COVID	3.73	1.50–9.25	0.005^**^
Perception: worse since COVID	12.16	9.57–15.51	<0.001^**^
Emotionally affected			
Age	0.95	0.77–1.17	0.639
Gender	1.27	0.97–1.67	0.089
Accommodations	3.15	2.08–4.77	<0.001^***^
Perception: better since COVID	2.99	1.30–6.90	0.010^**^
Perception: worse since COVID	15.49	12.05–19.91	<0.001^***^

*OR, Odds Ratio; CI, Confidence Interval*.

* *Statistical significance (P-value < 0.05)*.

** *Statistical significance (P-value < 0.01)*.

*** *Statistical significance (P-value < 0.001)*.

#### Domain 1: Cognition

Participants who felt that the pandemic had worsened their ability to learn new tasks were 17.46 times more likely to report pre-existing difficulties with learning (*p* < 0.001, 95% CI: 13.46–22.62). Those requiring physical or psychological accommodations had greater odds of reporting difficulties concentrating (OR = 2.10; *p* < 0.001, 95% CI: 1.44–3.07). Likewise, those who perceive that their concentration has worsened since the pandemic were 18.5 times more likely to have a pre-existing poor concentration (*p* < 0.001, 95% CI: 14.03–24.27). Participants older than 45 had significantly lower odds of reporting difficulties learning new tasks (OR = 0.76; *p* = 0.02, 95% CI: 0.60–0.96). *Post-hoc* analysis revealed that increased age slightly decreased the odds of reporting difficulties learning new tasks (OR = 0.98; *p* < 0.05, 95% CI: 0.97–0.996).

#### Domain 2: Mobility

Participants over the age of 45 had greater odds of reporting difficulties standing for long periods (OR = 1.55; *p* = 0.001, 95% CI: 1.19–2.00) and walking long distances (OR = 1.59, *p* = 0.001; 95% CI: 1.20–2.09), respectively. *Post-hoc* analysis also revealed that increased age slightly increased the odds of reporting difficulties for these variables (OR = 1.04; *p* < 0.001, 95% CI: 1.02–1.05 and OR = 1.04; *p* < 0.001, 95% CI: 1.02–1.05). Respondents requiring accommodations had greater odds of reporting difficulties standing up (OR = 2.32, *p* < 0.001; 95% CI: 1.56–3.44) and walking long distances (OR = 3.33; *p* < 0.001, 95% CI: 2.24–4.95). Participants who reported that their response has worsened since the pandemic were 12.69 times more likely to have difficulties standing up (*p* < 0.001, 95% CI: 9.78–16.48) and 14.5 times more likely to have difficulties walking long distances (*p* < 0.001, 95% CI: 10.90–19.23).

#### Domain 3: Self-Care

Participants requiring accommodations had greater odds of reporting difficulties washing their body (OR = 1.97, *p* = 0.02; 95% CI: 1.10–3.55). Also, participants who perceived that the pandemic has worsened their symptoms reported 47.82 times more likely to have difficulties washing their bodies (*p* < 0.001; 95% CI: 28.83–79.32) and 29.24 times more likely to have difficulties getting dressed (*p* < 0.001; 95% CI: 17.78–48.13).

#### Domain 4: Getting Along

Respondents requiring accommodations had significantly greater odds of reporting difficulties dealing with others (OR = 1.88; *p* = 0.001, 95% CI: 1.28–2.75). Those who had felt the pandemic worsened their response was 17.46 times more likely to have difficulties dealing with people they did not know (*p* < 0.001, 95% CI: 13.19–23.12). Furthermore, participants who were older than 45 years had significantly lower odds of reporting difficulties maintaining friendships (OR = 0.67, *p* = <0.001; 95% CI: 0.54–0.84). *Post-hoc* analysis revealed that increased age mildly decreased the odds of reporting difficulties in maintaining friendships (OR = 0.98; *p* < 0.05, 95% CI: 0.97–0.99). Those perceiving that the pandemic has worsened their symptoms had greater odds of difficulties maintaining friendships (OR = 14.35; *p* < 0.001; 95% CI: 10.81–19.04).

#### Domain 5: Life Activities

There was no statistical difference between those above or below the age of 45. Exploring age as a continuous variable, we discovered a modest correlation suggesting that increased age decreased the risk of having challenges in terms of taking care of household responsibilities (OR = 0.98, *p* < 0.05, 95% CI: 0.97–0.99). Furthermore, participants over the age of 45 had significantly lower odds of reporting difficulties performing day-to-day work (OR = 0.75; *p* = 0.004, 95% CI: 0.61–0.91). *Post-hoc* analysis revealed that increased age mildly decreased the odds of reporting difficulties performing day-to-day work (OR = 0.98; *p* < 0.05, 95% CI: 0.97–0.99). Women had significantly greater odds of reporting difficulties taking care of household responsibilities (OR = 1.68; *p* < 0.001, 95% CI: 1.29–2.19). Participants who required accommodations had significantly greater odds reporting difficulties taking care of household responsibilities (OR = 1.67; *p* = 0.008, 95% CI: 1.143–2.43) and performing day-to-day work (OR = 1.57; *p* = 0.017, 95% CI: 1.09–2.26). Perceiving that COVID-19 has worsened their symptoms increased the odds of having difficulties in taking care of household responsibilities (OR = 11.67; *p* < 0.001, 95% CI: 9.33–14.60) and completing day-to-day work (OR = 15.61; *p* < 0.001; 95% CI: 11.68–20.85).

#### Domain 6: Participation

Respondents who perceived more difficulties since the pandemic were more likely to have challenges in joining community activities (OR: 12.16; *p* = 0.005, 95% CI: 9.57–15.51) and were 15.49 times more likely to be affected by other health problems (*p* < 0.001, 95% CI: 12.05–19.91). Furthermore, participants who required accommodations had significantly odds of reporting difficulties participating in community activities (OR: 1.66; *p* = 0.006, 95% CI: 1.16–2.38) and being emotionally affected by other health problems (OR = 3.15; *p* < 0.001; 95% CI = 2.08–4.77).

## Discussion

We examined the perceived impact of the pandemic on functional activities of education workers in Ontario, Canada using the WHODAS 2.0 SF. The WHODAS 2.0 SF addresses difficulties due to health conditions; it provides a measure of disability under the ICIDH-2 framework in which disabilities arise when difficulties with form or function prevent desired levels of participation in society. Disability measured in this way reflects both relatively objective and reliable difficulties workers face. It also provides some guidance as to the levels of accommodation, which could potentially be required as a matter of policy.

To our knowledge, this is the first study to explore this area among education workers. Cross-sectional surveys are inherently limited in their capacity to investigate cause and effect. However, the salience of COVID-19 and related public health measures gives confidence that participants can generally attribute changes in their functional capacity to this period. Overall, education workers perceived that their capacities for functional activities have worsened since the pandemic.

A key finding in the present study is that there are associations between how individuals perceived the impact of COVID-19 and functional activity ratings. These associations were evident across all six domains, an essential consideration for school employers, policymakers, and rehabilitation researchers. Several reasons could explain how the pandemic influenced functional activities. For instance, it could be due to the challenges of setting boundaries between work and home life (16). While most Ontario workers were physically at work, there is naturally more reliance on technology to complete day-to-day tasks, including meetings and the stress of the hybrid model. Thus, we suspect that establishing boundaries between work and home duties is a contributor. Furthermore, with the COVID restrictions, it is unsurprising to find challenges in domains such as participation and getting along. However, what is critical from a policy and employment perspective is that the impact of COVID-19 falls most strongly on people who have pre-existing functional limitations. Therefore, planning for these difficulties and review of accommodations should be given some priority in the future.

The pandemic restrictions might have reduced mobility among some participants, especially older adults. Specifically, with prolonged inactivity and increased stress, mobility could be affected due to reduced muscle activity (17). Furthermore, factors such as fear of contamination, limited in-person socialization, and closures of fitness facilities could have affected education workers' mental wellbeing. Poor mental health and functional limitations potentially reinforce each other. This is concerning since depression and anxiety symptoms have negative implications across all six domains (6), and teachers' mental health is clearly at risk. These are important considerations and contribute to our understanding of the impact of COVID-19 on education workers' physical and mental wellbeing. It is also important to consider the potential long-term impact of the restriction measures on functional activities, including physical and cognitive impairments, because functional difficulties that are not addressed may in turn lead to difficulty managing disability and increased health care costs (18).

Older employees (i.e., ≥45) were more likely to have difficulties in mobility than younger employees. However, older age decreased the odds of adverse outcomes for some WHODAS domains. They were less likely to report difficulties learning new tasks (cognition) and maintaining friendships (getting along). While evidence suggests that older education workers had more difficulties adapting to some aspects of their jobs, such as technology, they were more eager to advance their knowledge than younger employees (19). Notably, younger participants were more likely to be impacted by COVID-19. This could be due to poorer coping abilities to deal with the consequences of the pandemic despite having more access to social support (6).

Age as a continuous variable produced relatively similar results as dichotomizing age, although the correlations were relatively weak. This is because increased or decreased risk is not entirely linear. Specifically, significant changes in scores changes were more visible in older age groups instead of a steady change in score year by year.

Our results revealed that women were more likely to have difficulties taking care of household responsibilities than men. Some evidence suggests that women tend to be more involved in household chores than men (20). However, a recent meta-analysis revealed that gender differences in work-life conflict are generally small (21). Another possible explanation could be the gender difference in the likelihood of reporting physical or psychosocial symptoms. Specifically, while men and women could exhibit similar symptoms, women were more likely to report their symptoms than men (22).

Individuals requiring accommodations were more likely to have difficulties in all functional areas. This could be due to difficulties managing their health and are often affected by work-related aspects such as stress, high workload, hostile interpersonal relationships, and dealing with strangers (23). While Ontario schools are compliant with the Accessibility for Ontarians with Disabilities Act (24), these workers are particularly vulnerable to negative treatment in the workplace, while issues around adequate resources and accessibility remain problematic pre-pandemic (25). We also found that employees requiring accommodations were more likely to be affected by their health problems. We suspect that the pandemic has likely exacerbated these concerns due to limited training or sufficient resources.

### Limitations

There were some limitations in this study. First, the cross-sectional nature of the questionnaire only examines a point in time and cannot be used to establish causal relationships. While we attempted to understand how workers fared before the pandemic, longitudinal research is needed to examine the impact of COVID-19 on activity limitations over extended periods. A second limitation was our inability to calculate an accurate response rate. Specifically, we could not confirm that all district leaders disseminated the survey links or if the members received the links. Other factors that may have reduced participation rates include the survey length since, as previously noted, the survey contained other outcomes beyond the scope of this study. Naturally, longer surveys have lower completion rates than shorter surveys. Furthermore, education workers could likely be experiencing research and pandemic fatigue (26, 27). Finally, despite our inability to accurately calculate a response rate, one must consider the challenging climate some employees faced during that period. Thus, they could be less inclined to participate in COVID-related studies. Nevertheless, this study offers how participants perceived how the pandemic has impacted their functional activities. Accordingly, we believe these results remain essential for occupational, research, and policy considerations.

### Recommendations

Our findings support the argument that education workers face challenges during the pandemic. Accordingly, improving working conditions in educational settings is essential. To mitigate the harmful effects of COVID-19 and associated public health measures, school policies must focus on promoting employees' wellbeing. Policymakers should consider the impact of COVID-19, including provincial restrictions on education workers with a health promotion lens. This is a complex undertaking as safety (i.e., infections) must remain a priority, as they play a significant role in supporting a vulnerable population, including disadvantaged children, students with special needs, and poor mental health.

Individuals suffering from poorer mental health, affecting their daily functions due to the pandemic restrictions, may benefit from telehealth services without requiring face-to-face contact. Overall, telehealth services help maintain patients' physical and psychosocial health while without the risk of contagion (28). Typically, permanent employees in Ontario receive employee and family assistance programs from their employers. Thus, employers should remind employees of these services and offer support on accessing such services.

School administrators should provide adequate training for education workers to improve their technological skills and virtual competence. Müller et al. (29) found that educators perceived less stress after receiving training in online teaching platforms. From a social perspective, online social events were shown to reduce stress among educators (16). Therefore, virtual social events when in-person social gatherings are not feasible could be helpful.

Recognizing the possible obstacles employees with accommodations could be facing during the pandemic, employers should offer a more tailored approach to address their needs. These employees should also be involved in implementing policies affecting their work, as previous research suggests limited involvement pre-pandemic (30). Finally, from a research perspective, researchers should examine employees' experiences with various disabilities during the pandemic to better understand their needs.

## Conclusions

This study offers insight into the perceived impact of COVID-19 on functional activities in educational workers in Ontario, Canada. Overall, employees perceived worse functional activities since the pandemic. Furthermore, those requiring accommodations have worse functional outcomes. Despite provincial mandates to support those with disabilities, more research is required to understand the needs of education workers requiring accommodations within the context of the pandemic. Older participants had poorer mobility outcomes; however, they appeared to have better coping skills in learning new tasks and maintaining friendships. Furthermore, women had greater odds of experiencing difficulties in maintaining household responsibilities. Based on the results, we suspect that restrictions to reduce the spread of the virus have contributed to mobility, getting along, participation, and life activities. Also, due to the restrictions, we suspect that poorer mental health outcomes also affect one's abilities in all six domains. Based on these findings, we suggest that policymakers incorporate a health promotion lens to support their employees, including tailored support for employees requiring accommodations.

## Data Availability Statement

The datasets presented in this article are not readily available because of ethical restrictions. Requests to access the datasets should be directed to the corresponding author.

## Ethics Statement

The studies involving human participants were reviewed and approved by Stephen P. Lewis, University of Guelph. The patients/participants provided their written informed consent to participate in this study.

## Author Contributions

Material preparation and data collection were performed by FS, BG, BN-K, RB, and JC. Analysis was performed by BG, FS, and BO. The first draft of the manuscript was written by FS. All authors edited subsequent versions of the manuscript. All authors contributed to the study's conception and design, read, and approved the final manuscript.

## Funding

The Centre for Research in Occupational Safety and Health funded the article processing charge of this manuscript.

## Conflict of Interest

The authors declare that the research was conducted in the absence of any commercial or financial relationships that could be construed as a potential conflict of interest.

## Publisher's Note

All claims expressed in this article are solely those of the authors and do not necessarily represent those of their affiliated organizations, or those of the publisher, the editors and the reviewers. Any product that may be evaluated in this article, or claim that may be made by its manufacturer, is not guaranteed or endorsed by the publisher.
